# Social determinants of denture/bridge use: Japan gerontological evaluation study project cross-sectional study in older Japanese

**DOI:** 10.1186/1472-6831-14-63

**Published:** 2014-06-03

**Authors:** Tatsuo Yamamoto, Katsunori Kondo, Jun Aida, Kayo Suzuki, Jimpei Misawa, Miyo Nakade, Shinya Fuchida, Yukio Hirata

**Affiliations:** 1 Department of Dental Sociology, Kanagawa Dental University Graduate School of Dentistry, 82 Inaoka-cho, Yokosuka, Kanagawa 238-8580, Japan; 2 Center for Preventive Medical Science, Chiba University, Chiba, Japan; 3 Center for Well-being and Society, Nihon Fukushi University, Nagoya, Japan; 4 Department of International and Community Oral Health, Tohoku University Graduate School of Dentistry, Sendai, Japan; 5 Department of Policy Studies, Aichi Gakuin University, Nisshin, Japan; 6 Faculty of Sociology, Rikkyo University, Tokyo, Japan; 7 Department of Nutrition, Faculty of Health and Nutrition, Tokaigakuen University, Nagoya, Japan

**Keywords:** Social determinants, Dental prosthesis, Older people, Cross-sectional study

## Abstract

**Background:**

Studies suggest that using a denture/bridge may prevent disability in older people. However, not all older people with few remaining teeth use a denture/bridge. This cross-sectional study aimed to examine the social determinants which promote denture/bridge use among older Japanese.

**Methods:**

A total of 54,388 (25,630 males and 28,758 females) community-dwelling individuals aged 65 or over, living independently, able to perform daily activities, and with 19 or fewer teeth. The dependent variable was denture/bridge use. Socio-demographics, number of teeth, present illness, social participation, social support, and social networks were used as individual-level independent variables. Data for social capital were aggregated and used as local district (n = 561 for males, n = 562 for females) -level independent variables. Number of dentists working in hospitals/clinics per population and population density were used as municipality (n = 28) -level independent variables. Three-level multilevel Poisson regression analysis was performed for each sex.

**Results:**

High equivalent income, low number of teeth, present illness, and living in a municipality with high population density were significantly associated with denture/bridge use in both sexes in the fully adjusted models (*p* < 0.05). Denture/bridge use was significantly associated with high educational attainment in males and participating in social groups in females in the fully adjusted model (*p* < 0.05). No significant associations were observed between denture/bridge use and social capital.

**Conclusions:**

Denture/bridge use was significantly associated with high economic status and present illness in both sexes, high educational attainment in males, and participation in social groups in females among community-dwelling older Japanese after adjusting for possible confounders.

## Background

Fixed and removable prostheses are most commonly used to replace missing teeth with the aim of improving chewing ability, aesthetics and pronunciation. Recent studies have reported favorable effects of prosthodontic treatment on systemic health [1-4]. An intervention study showed that prosthodontic treatment improved the nutritional status of institutionalized older people [1]. Cohort studies have reported that older people who do not use dentures despite having few remaining teeth show a higher risk of dementia onset [2] and incident falls [3] after adjusting for possible confounders. Moreover, these studies showed no significant difference in dementia onset and incident falls between subjects having few teeth and using dentures and those having 20 or more teeth, suggesting that denture use may reduce the risk of dementia onset and incident falls in subjects having few teeth. Another cohort study showed that the use of dentures is associated with a decreased risk of mortality in edentulous older people [4].

Despite these favorable effects of dentures and bridges, not all persons who lose their teeth use a denture/bridge. In a 2011 Japanese national survey, approximately 29%, 19%, and 14% of the whole population aged 65–69, 70–74, and 75–79 did not use a denture/bridge, respectively [5]. A study from the UK reported that 25% and 15% of patients who were provided with partial dentures never or only occasionally wore dentures, respectively [6].

Denture/bridge use reflects access to dental care. Many factors have been reported to directly and indirectly influence utilization of dental services in older people. These factors can be divided into four main categories: socio-demographic factors, ill health-related factors, service-related factors, and attitude or subjective factors [7]. Socio-demographic factors include sex, age, education, and income [8,9]. Ill health-related factors include number of teeth, general ill health, and functional limitations [10,11]. Service-related factors include accessibility and insurance coverage [8,9,12]. Attitude or subjective factors include personal beliefs and satisfaction with dental visits [13,14]. These factors may be associated with denture/bridge use.

Little is known about the factors associated with denture/bridge use. A study among older people in a Japanese municipality revealed an association between denture/bridge use and economic status [15]. An exploratory qualitative interview study from the UK reported differences in the attitudes of dentists and patients to the provision of removable partial dentures [16]. Dentists focused on restoration of physical function of the teeth, whereas patients focused on the social significance of oral rehabilitation.

It has previously been reported that access to medical care may be associated with social relationships and social capital [17]. However, the association between access to dental care and denture/bridge use and social relationships is unknown, although previous studies have suggested an association between dental health and social relationships [18,19]. Individuals obtain health information through their connections with others, and it is possible that this may lead to denture/bridge use. Individuals with few teeth may need to wear denture/bridge to improve appearance or speech, which would help them interact in groups.

The purpose of this study was to examine the social determinants which promote denture/bridge use using cross-sectional data from community-dwelling older Japanese people. First, the association of denture/bridge use with known factors associated with access to dental care (socio-demographic factors, ill health-related factors, and service-related factors) was examined. Then, taking into account these factors, the association with denture/bridge use and social relationship factors was analyzed. In particular, multilevel (first level, individual; second level, local district; and third level, municipality) analysis was used because it is appropriate to assess contextual and individual determinants of health outcomes, which were not assessed in previous studies on factors associated with access to dental care and denture/bridge use [7-16].

## Methods

### Study population

Data from a cross-sectional study, which were collected as part of the Japan Gerontological Evaluation Study (JAGES) Project, were used for this on-going Japanese prospective cohort study. JAGES aims to conduct empirical studies from gerontological and social epidemiological perspectives. The sample was restricted to those who did not already have a physical or cognitive disability, defined by not receiving public long-term care insurance benefits, at baseline. From July 2010 to January 2012, a mail survey was conducted in a random sample of 169,215 community-dwelling individuals aged 65 years or over residing in 31 municipalities in 12 prefectures in Japan. Of the 112,123 respondents (response rate, 66.3%), 8,502 subjects were excluded due to a lack of age and sex information. Then, 5,851 subjects in three municipalities were excluded due to a lack of information on local district, social support, and/or general trust. After excluding 411 (lack of information of local district) and 4,525 (status of activities of daily living (ADL) was dependent or unknown) subjects, a total of 92,834 subjects aged 65 or older in 562 local districts in 28 municipalities were included in the present study. The JAGES protocol was reviewed and approved by the Ethics Committee on Research of Human Subjects at Nihon Fukushi University.

### Outcome variables

Dental status was assessed using a self-administered questionnaire [20]. Respondents were asked to classify their dental status as having 20 or more, 10–19, 1–9 or 0 teeth. Data from all subjects, including those having 20 or more teeth, were used when local district-level social capital was calculated. However, subjects having 20 or more teeth were excluded when univariate and multilevel prevalence ratios (PRs) were calculated. Denture/bridge use was ascertained by asking, “Do you use a denture or bridge?” with possible answers dichotomized into yes and no.

### Socio-demographic variables

Data on socio-demographics (sex, age, marital status, educational attainment, equivalent income) were obtained using a self-administered questionnaire. To adjust household income for household size, equivalent income was calculated by dividing the household income by the square root of the number of household members, and placed into one of seven categories (<500,000 yen, 500,000-999,999 yen, 1,000,000-1,499,999 yen, 1,500,000-1,999,999 yen, 2,000,000-2,999,999 yen, 3,000,000-3,999,999 yen, and ≥4,000,000 yen).

### Ill health-related variables

Number of teeth and present illness were considered as ill health-related variables. Self-reported current medical treatment for cancer, heart disease, stroke, hypertension, diabetes, obesity, hyperlipidemia, osteoporosis, arthritis, trauma, respiratory disease, gastrointestinal disease, liver disease, mental illness, visual/hearing impairment, dysphagia, urinary disease, sleep disorder, or other conditions was used as the variable present illness, dichotomized into yes and no.

### Service-related variables

Data on the number of dentists working in hospitals or clinics were obtained from the Survey of Physicians, Dentists and Pharmacists conducted by the Ministry of Health, Labour and Welfare, Japan in 2010. Data on population in 2010 and area of inhabitable land of each municipality were obtained from the National Population Census Survey conducted by the Ministry of Internal Affairs and Communications, Japan. Number of dentists working in hospitals or clinics per 100,000 people and population density were calculated for each municipality. The number of dentists working in hospitals or clinics per 100,000 people was categorized into four groups (lowest, low middle, high middle, or highest) based on 25th, 50th, and 75th percentiles. Population density was categorized into four groups (metropolitan, urban, semi-urban, or rural-agricultural).

### Social relationship variables

General trust, norms of reciprocity, and attachment to place were assessed by asking “Generally speaking, would you say that most people can be trusted?”, “Would you say that most of the time people try to be helpful?”, and “Do you feel attached to the area you live?” with possible answers dichotomized into yes and no (including “depends”). For social participation, respondents were asked whether they belonged to industrial and trade associations, volunteer groups, older people’s clubs, sports groups or clubs, neighborhood associations or councils, or hobby clubs, with possible answers dichotomized into yes and no. The number of social groups was calculated for each subject.

Emotional and instrumental social support, both received and given, was evaluated by using the following questions: “Do you have someone who listens to your concerns and complaints?” (emotional social support received), “Do you listen to someone’s concerns and complaints?” (emotional social support given), “Do you have someone who looks after you when you are sick and have to stay in bed for a few days?” (instrumental social support received), and “Do you look after someone when he/she is sick and stays in bed for a few days?” (instrumental social support given), with possible answers dichotomized into yes and no.

Social network was measured by the question, “How often do you see your friends?” with the following possible answers: “almost every day”, “two or three times per week”, “once a week”, “once or twice per month”, “several times per year”, or “rarely”.

We created local district (n = 561 for males, n = 562 for females) -level social capital variables by aggregating the individual-level data on general trust, norms of reciprocity, attachment to place, social support (both emotional and instrumental received and given), number of social groups, and meeting friends (% of subjects meeting friends at least several times a year). General trust, norms of reciprocity, attachment to place, and social support were categorized as cognitive social capital. Number of social groups and meeting friends were categorized as structural social capital. Local districts were categorized into four groups (lowest, low middle, high middle, or highest) based on 25th, 50th, and 75th percentiles for each variable.

### Analysis

The following analyses were conducted in subjects with 19 or fewer teeth (25,630 males and 28,758 females). First, univariate PRs and 95% confidence intervals (CIs) were calculated for each independent variable with denture/bridge use as the dependent variable in each sex. Because the percentage of people using a denture/bridge was high (males: 68.1%, females: 67.6%), adjusted odds ratio derived from the logistic regression could no longer approximate PR [21]. Therefore, multilevel Poisson regression model with random intercepts and fixed slopes was used separately for males and females to calculate multilevel PRs, taking into account variations in the outcomes between local districts and municipalities using MLwiN 2.28 (Centre for Multilevel Modelling, University of Bristol, Bristol, UK), with denture/bridge use as the dependent variable [22]. In model 1, socio-demographics (age, marital status, educational attainment, and equivalent income), health status (number of teeth and present illness) and municipality-level characteristics (number of dentists working in hospitals or clinics per 100,000 people and population density) were added. In models 2 and 3, number of social groups and frequency of meeting friends, both of which were significantly associated with denture/bridge use in the previous univariate analysis, were added to model 1, respectively. Moreover, to examine the association between each local district-level social capital variable and denture/bridge use after adjusting for socio-demographics, health status and municipality-level characteristics, each local district-level social capital variable was added to model 1. In the model, corresponding individual level variable was also added to avoid ecological fallacy.

## Results

The percentages of males and females using a denture/bridge were 68.1% and 67.6%, respectively. Table 1 shows the PRs (95% CIs) for denture/bridge use according to individual-level variables. In both sexes, high equivalent income, low number of teeth, present illness, involvement in two or more kinds of social groups, and meeting friends 1–2 times per month were significantly associated with denture/bridge use. Age group, marital status, educational attainment, and instrumental social support given were associated with denture/bridge use in males.

**Table 1:** Association between denture/bridge use and individual-level characteristics in males and females

	**Males, n = 25630**				**Females, n = 28758**			
	**n**	**Denture/bridge users (%)**	**Univariate PR**		**n**	**Denture/bridge users (%)**	**Univariate PR**	
**Characteristic**			**PR**	**95% CI**			**PR**	**95% CI**
Socio-demographics								
Age group (years)								
65 - 69	6699	69.7	1.00	(reference)	6923	68.5	1.00	(reference)
70 - 74	7081	68.1	0.98	(0.94-1.02)	7916	66.2	0.97	(0.93-1.01)
75 - 79	6118	66.5	0.95	(0.92-0.99)^a^	6880	67.5	0.99	(0.95-1.03)
80 - 84	3899	68.2	0.98	(0.93-1.03)	4443	68.2	1.00	(0.95-1.04)
≥ 85	1833	67.9	0.97	(0.92-1.04)	2596	69.3	1.01	(0.96-1.07)
Marital status								
Married	21449	68.7	1.00	(reference)	15547	67.3	1.00	(reference)
Separated/divorced	3216	65.9	0.96	(0.92-1.00)	11868	68.6	1.02	(0.99-1.05)
Never married	441	60.5	0.88	(0.78-0.99)^a^	604	66.2	0.98	(0.89-1.09)
Unknown/missing	524	63.9	0.93	(0.83-1.04)	739	59.8	0.89	(0.81-0.98)^a^
Educational attainment (years)								
< 6	453	62.7	1.00	(reference)	1028	66.4	1.00	(reference)
6 - 9	11161	62.6	1.00	(0.89-1.12)	13582	65.2	0.98	(0.91-1.06)
10 - 12	7877	70.7	1.13	(1.00-1.27)^a^	9512	70.2	1.06	(0.98-1.14)
≥ 13	5048	77.4	1.23	(1.10-1.39)^b^	3171	70.7	1.06	(0.98-1.16)
Missing	1091	66.0	1.05	(0.92-1.21)	1465	67.8	1.02	(0.93-1.12)
Equivalent income (10000 yen)								
< 50	739	60.1	1.00	(reference)	1625	60.9	1.00	(reference)
50 - 99	2192	59.6	0.99	(0.89-1.10)	3274	65.0	1.07	(0.99-1.15)
100 - 149	3214	62.2	1.04	(0.93-1.15)	3151	66.4	1.09	(1.01-1.18)^a^
150 - 200	5245	68.5	1.14	(1.03-1.26)^b^	4294	68.2	1.12	(1.04-1.20)^b^
200 - 299	5452	71.6	1.19	(1.08-1.32)^c^	4768	71.5	1.18	(1.09-1.26)^c^
300 - 399	3275	75.6	1.26	(1.14-1.39)^c^	2760	74.5	1.22	(1.13-1.32)^c^
≥ 400	2266	75.2	1.25	(1.13-1.39)^c^	2226	73.1	1.20	(1.11-1.30)^c^
Missing	3247	62.8	1.05	(0.94-1.16)	6660	63.4	1.04	(0.97-1.12)
Health status								
Number of teeth								
10 - 19	10407	64.9	1.00	(reference)	11129	62.1	1.00	(reference)
1 - 9	9786	73.5	1.13	(1.09-1.17)^c^	11565	73.7	1.19	(1.15-1.22)^c^
0	5437	64.5	0.99	(0.95-1.04)	6064	66.4	1.07	(1.03-1.11)^c^
Present illness								
No	6192	65.1	1.00	(reference)	6214	65.0	1.00	(reference)
Yes	17602	69.2	1.06	(1.03-1.10)^c^	20081	68.2	1.05	(1.01-1.09)^b^
Missing	1836	68.4	1.05	(0.99-1.12)	2463	69.7	1.07	(1.01-1.13)^a^
Social relationship								
General trust								
No	1075	64.6	1.00	(reference)	1273	64.8	1.00	(reference)
Yes	23326	68.3	1.06	(0.98-1.14)	26111	67.8	1.05	(0.98-1.12)
Missing	1229	68.1	1.05	(0.95-1.17)	1374	66.8	1.03	(0.94-1.13)
Norms of reciprocity								
No	2237	66.5	1.00	(reference)	2517	66.9	1.00	(reference)
Yes	22089	68.4	1.03	(0.98-1.08)	24635	67.8	1.01	(0.96-1.07)
Missing	1304	66.9	1.01	(0.92-1.09)	1606	67.1	1.00	(0.93-1.08)
Attachment to place								
No	1219	67.8	1.00	(reference)	1285	65.8	1.00	(reference)
Yes	23754	68.2	1.01	(0.94-1.08)	26631	67.7	1.03	(0.96-1.10)
Missing	657	67.0	0.99	(0.88-1.11)	842	68.1	1.03	(0.93-1.15)
Emotional social support (received)								
No	1211	64.9	1.00	(reference)	727	64.1	1.00	(reference)
Yes	11573	66.7	1.03	(0.96-1.10)	12619	65.7	1.03	(0.93-1.13)
Missing	12846	69.8	1.07	(1.00-1.16)	15412	69.4	1.08	(0.99-1.19)
Emotional social support (given)								
No	2225	65.0	1.00	(reference)	1112	64.2	1.00	(reference)
Yes	13112	67.3	1.03	(0.98-1.09)	12703	65.8	1.02	(0.95-1.11)
Missing	10293	70.0	1.08	(1.02-1.14)^b^	14943	69.5	1.08	(1.00-1.17)^a^
Instrumental social support (received)								
No	1245	65.1	1.00	(reference)	1380	63.9	1.00	(reference)
Yes	15949	67.7	1.04	(0.97-1.12)	16461	66.9	1.05	(0.98-1.12)
Missing	8436	69.3	1.07	(0.99-1.15)	10917	69.3	1.08	(1.01-1.16)^a^
Instrumental social support (given)								
No	2929	64.8	1.00	(reference)	3855	66.7	1.00	(reference)
Yes	14467	68.2	1.05	(1.00-1.11)^a^	13287	66.6	1.00	(0.96-1.04)
Missing	8234	69.1	1.07	(1.01-1.12)^a^	11616	69.1	1.04	(0.99-1.08)
Number of social groups								
0	6074	66.4	1.00	(reference)	6693	66.5	1.00	(reference)
1	4390	68.5	1.03	(0.99-1.08)	4551	69.9	1.05	(1.01-1.10)^a^
2	3355	70.5	1.06	(1.01-1.12)^a^	3306	70.2	1.06	(1.00-1.11)^a^
3 - 6	5207	71.1	1.07	(1.02-1.12)^b^	3871	70.2	1.06	(1.01-1.11)^a^
Missing	6604	66.0	0.99	(0.95-1.04)	10337	65.6	0.99	(0.95-1.03)
Frequency of meeting friends								
Rarely	2866	64.5	1.00	(reference)	1891	64.5	1.00	(reference)
Several times a year	5163	69.5	1.08	(1.02-1.14)^b^	3127	68.7	1.07	(0.99-1.14)
1 or 2 times/month	4986	69.7	1.08	(1.02-1.14)^b^	5225	69.1	1.07	(1.00-1.14)^a^
Once/week	3595	69.1	1.07	(1.01-1.14)^a^	4965	68.2	1.06	(0.99-1.13)
2 or 3 times/week	4304	67.6	1.05	(0.99-1.11)	6979	67.2	1.04	(0.98-1.11)
Almost everyday	2930	68.2	1.06	(0.99-1.13)	4159	67.5	1.05	(0.98-1.12)
Missing	1786	65.3	1.01	(0.94-1.09)	2412	65.8	1.02	(0.95-1.10)

PR, prevalence ratio; CI, confidence interval.

^a^, *p* < 0.05; ^b^, *p* < 0.01; ^c^, *p* < 0.001.

Table 2 shows the PRs (95% CIs) for denture/bridge use according to local district- and municipality-level variables. Local district-level emotional and instrumental social support received, meeting friends, and municipality-level population density were significantly associated with denture/bridge use in both sexes. Municipality-level number of dentists working in hospitals or clinics per population was associated with denture/bridge use in males, and emotional social support given in females.

**Table 2:** Association between denture/bridge use and local district- and municipality-level characteristics in males and females

	**Males**				**Females**			
	**n**	**Denture/bridge users (%)**	**Univariate PR**		**n**	**Denture/bridge users (%)**	**Univariate PR**	
**Characteristic**			**PR**	**95% CI**			**PR**	**95% CI**
Local district-level characteristics								
Cognitive social capital								
General trust (%)								
Lowest (<92.86)	85	68.6	1.00	(reference)	85	67.0	1.00	(reference)
Low middle (92.86 - 97.00)	304	67.8	0.99	(0.93-1.05)	304	67.5	1.01	(0.95-1.07)
High middle (97.01 - 99.99)	114	69.2	1.01	(0.94-1.08)	114	68.5	1.02	(0.96-1.09)
Highest (100.00)	58	69.8	1.02	(0.91-1.13)	59	68.2	1.02	(0.92-1.13)
Norms of reciprocity (%)								
Lowest (<84.91)	61	71.1	1.00	(reference)	61	68.0	1.00	(reference)
Low middle (84.91 - 91.17)	250	69.3	0.98	(0.90-1.06)	250	68.8	1.01	(0.93-1.10)
High middle (91.18 - 99.99)	237	67.0	0.94	(0.87-1.02)	234	66.7	0.98	(0.90-1.06)
Highest (100.00)	13	71.6	1.01	(0.77-1.32)	14	69.7	1.02	(0.81-1.30)
Attachment to place (%)								
Lowest (<91.03)	62	70.4	1.00	(reference)	63	68.3	1.00	(reference)
Low middle (91.03 - 97.11)	379	68.4	0.97	(0.90-1.05)	379	67.9	0.99	(0.92-1.07)
High middle (97.12 - 99.99)	84	66.4	0.94	(0.87-1.03)	84	66.3	0.97	(0.89-1.05)
Highest (100.00)	36	66.7	0.95	(0.81-1.10)	36	70.3	1.03	(0.89-1.18)
Emotional social support (received) (%)								
Lowest (<88.89)	142	71.9	1.00	(reference)	143	71.2	1.00	(reference)
Low middle (88.89 - 92.85)	139	69.7	0.97	(0.92-1.02)	139	69.5	0.98	(0.93-1.03)
High middle (92.86 - 95.44)	139	67.5	0.94	(0.89-0.99)^a^	139	67.5	0.95	(0.90-1.00)^a^
Highest (≥95.45)	138	65.7	0.91	(0.86-0.97)^b^	138	64.3	0.90	(0.86-0.95)^c^
Missing	3	45.6	0.63	(0.49-0.83)^c^	3	59.6	0.84	(0.69-1.01)
Emotional social support (given) (%)								
Lowest (<85.71)	129	69.4	1.00	(reference)	130	70.4	1.00	(reference)
Low middle (85.71 - 89.35)	151	69.0	0.99	(0.94-1.04)	151	67.8	0.96	(0.92-1.01)
High middle (89.36 - 92.09)	140	66.9	0.96	(0.91-1.01)	140	66.3	0.94	(0.90-0.99)^a^
Highest (≥92.10)	141	68.7	0.99	(0.94-1.04)	141	68.5	0.97	(0.92-1.03)
Instrumental social support (received) (%)								
Lowest (<88.89)	148	71.2	1.00	(reference)	148	70.4	1.00	(reference)
Low middle (88.89 - 92.26)	133	66.8	0.94	(0.89-0.99)^a^	133	66.7	0.95	(0.90-1.00)^a^
High middle (92.27 - 94.81)	140	68.4	0.96	(0.91-1.01)	140	67.9	0.97	(0.92-1.01)
Highest (≥94.82)	140	67.8	0.95	(0.90-1.00)	141	67.1	0.95	(0.91-1.00)
Instrumental social support (given) (%)								
Lowest (<77.42)	140	67.9	1.00	(reference)	141	69.1	1.00	(reference)
Low middle (77.42 - 81.87)	140	67.6	1.00	(0.95-1.05)	140	66.7	0.97	(0.92-1.01)
High middle (81.88 - 85.41)	141	67.5	0.99	(0.95-1.04)	141	67.5	0.98	(0.93-1.02)
Highest (≥85.42)	140	70.3	1.03	(0.98-1.09)	140	68.4	0.99	(0.94-1.04)
Structural social capital								
Mean number of social groups								
Lowest (<1.280)	140	69.2	1.00	(reference)	141	70.4	1.00	(reference)
Low middle (1.280 - 1.483)	141	69.4	1.00	(0.95-1.06)	141	69.2	0.98	(0.94-1.03)
High middle (1.484 - 1.687)	139	68.1	0.98	(0.94-1.03)	139	67.7	0.96	(0.92-1.01)
Highest (≥1.688)	141	67.0	0.97	(0.92-1.02)	141	65.6	0.93	(0.89-0.97)^b^
Meeting friends (%)								
Lowest (<87.76)	136	69.8	1.00	(reference)	137	70.2	1.00	(reference)
Low middle (87.76 - 91.00)	142	68.6	0.98	(0.93-1.04)	142	68.8	0.98	(0.93-1.03)
High middle (91.01 - 93.32)	137	68.8	0.98	(0.93-1.04)	137	67.3	0.96	(0.91-1.01)
Highest (≥93.33)	143	66.4	0.95	(0.90-1.00)	143	66.4	0.95	(0.90-1.00)^a^
Missing	3	45.6	0.65	(0.50-0.85)^b^	3	59.6	0.85	(0.70-1.03)
Municipality-level characteristics								
Number of dentists per 100000 people								
Lowest (<47.29)	7	65.3	1.00	(reference)	7	65.7	1.00	(reference)
Low middle (47.29 - 53.97)	7	66.4	1.02	(0.96-1.07)	7	67.5	1.03	(0.98-1.08)
High middle (53.98 - 59.74)	7	67.7	1.04	(0.99-1.09)	7	67.4	1.03	(0.98-1.08)
Highest (≥59.75)	7	70.1	1.07	(1.02-1.13)^b^	7	68.5	1.04	(1.00-1.09)
Population density (/km^2^)								
Rural-agricultural (<1000)	2	64.4	1.00	(reference)	2	64.6	1.00	(reference)
Semi-urban (1000–1499)	7	68.1	1.06	(1.02-1.10)^b^	7	68.4	1.06	(1.02-1.10)^b^
Urban (1500–3999)	6	70.6	1.10	(1.05-1.14)^c^	6	68.5	1.06	(1.02-1.10)^b^
Metropolitan (≥4000)	13	72.2	1.12	(1.08-1.17)^c^	13	72.0	1.12	(1.07-1.16)^c^

PR, prevalence ratio; CI, confidence interval.

n, number of local districts for local district-level characteristics and number of municipalities for municipality-level characteristics.

^a^, *p* < 0.05; ^b^, *p* < 0.01; ^c^, *p* < 0.001.

Table 3 shows the results of multilevel Poisson regression analyses. High educational attainment, high equivalent income, low number of teeth, present illness, and high population density were significantly associated with denture/bridge use in males (*p* < 0.05). High equivalent income, low number of teeth present, present illness, involvement in one or more social groups, and high population density were significantly associated with denture/bridge use in females (*p* < 0.05). In both the sexes, frequency of meeting friends was not significantly associated with denture/bridge use in model 3. Significance of the variables in model 1 did not change after adding the variable of frequency of meeting friends in both sexes.

**Table 3:** Multilevel prevalence ratios and 95% confidence intervals for denture/bridge use in males and females

	**Males**				**Females**			
	**Model 1**		**Model 2**		**Model 1**		**Model 2**	
	**PR**	**95% CI**	**PR**	**95% CI**	**PR**	**95% CI**	**PR**	**95% CI**
Fixed effects								
Individual-level characteristics								
Age group (years) (reference 65–69)								
70 - 74	0.98	(0.94-1.02)	0.98	(0.94-1.02)	0.96	(0.92-1.00)^a^	0.96	(0.92-1.00)
75 - 79	0.96	(0.92-1.00)	0.96	(0.92-1.00)	0.97	(0.93-1.01)	0.97	(0.93-1.02)
80 - 84	0.98	(0.94-1.03)	0.99	(0.94-1.04)	0.98	(0.93-1.03)	0.99	(0.94-1.04)
≥ 85	0.99	(0.93-1.06)	1.00	(0.93-1.06)	0.99	(0.93-1.05)	1.00	(0.94-1.06)
Marital status (reference married)								
Separated/divorced	0.97	(0.93-1.02)	0.97	(0.93-1.02)	1.02	(0.99-1.06)	1.02	(0.99-1.05)
Never married	0.91	(0.80-1.03)	0.91	(0.81-1.03)	0.98	(0.89-1.08)	0.98	(0.89-1.08)
Unknown/missing	1.00	(0.89-1.11)	1.00	(0.90-1.12)	0.93	(0.84-1.02)	0.93	(0.85-1.03)
Educational attainment (years) (reference <6)								
6 - 9	0.97	(0.86-1.10)	0.97	(0.86-1.10)	0.99	(0.92-1.08)	0.99	(0.92-1.08)
10 - 12	1.07	(0.95-1.21)	1.07	(0.95-1.21)	1.06	(0.98-1.15)	1.06	(0.97-1.15)
≥ 13	1.15	(1.02-1.30)^a^	1.15	(1.01-1.30)^a^	1.07	(0.98-1.17)	1.06	(0.97-1.16)
Missing	1.00	(0.87-1.15)	1.00	(0.87-1.15)	1.02	(0.92-1.13)	1.02	(0.92-1.13)
Equivalent income (10000 yen) (reference <50)								
50 - 99	0.99	(0.89-1.10)	0.99	(0.89-1.10)	1.06	(0.99-1.15)	1.06	(0.99-1.15)
100 - 149	1.02	(0.92-1.13)	1.02	(0.92-1.13)	1.08	(1.00-1.17)^a^	1.08	(1.00-1.16)
150 - 200	1.10	(0.99-1.21)	1.09	(0.99-1.21)	1.11	(1.03-1.20)^b^	1.10	(1.03-1.19)^b^
200 - 299	1.14	(1.03-1.25)^a^	1.13	(1.02-1.25)^a^	1.16	(1.08-1.24)^c^	1.15	(1.07-1.23)^c^
300 - 399	1.18	(1.07-1.31)^b^	1.18	(1.06-1.30)^b^	1.20	(1.12-1.30)^c^	1.19	(1.10-1.29)^c^
≥ 400	1.18	(1.06-1.31)^b^	1.17	(1.05-1.30)^b^	1.18	(1.09-1.27)^c^	1.17	(1.08-1.26)^c^
Missing	1.04	(0.93-1.15)	1.03	(0.93-1.15)	1.03	(0.96-1.11)	1.03	(0.96-1.11)
Number of teeth (reference 10–19)								
1 - 9	1.16	(1.13-1.20)^c^	1.16	(1.13-1.20)^c^	1.21	(1.17-1.25)^c^	1.21	(1.18-1.25)^c^
0	1.05	(1.01-1.10)^b^	1.05	(1.01-1.10)^a^	1.12	(1.08-1.17)^c^	1.13	(1.08-1.17)^c^
Present illness (reference no)								
Yes	1.06	(1.02-1.10)^b^	0.94	(0.91-0.98)^b^	1.05	(1.01-1.09)^b^	0.95	(0.92-0.99)^b^
Missing	1.05	(0.99-1.12)	0.99	(0.94-1.05)	1.08	(1.02-1.14)^b^	1.03	(0.98-1.08)
Number of social groups (reference 0)								
1			1.02	(0.97-1.07)			1.05	(1.01-1.10)^a^
2			1.03	(0.98-1.09)			1.05	(1.00-1.11)^a^
3 - 6			1.05	(1.00-1.10)			1.06	(1.01-1.11)^a^
Missing			1.02	(0.97-1.06)			1.00	(0.97-1.04)
Municipality-level characteristics								
Number of dentists per 100000								
people (reference lowest (<47.29))								
Low middle (47.29 - 53.97)	1.03	(0.98-1.09)	1.03	(0.98-1.09)	1.04	(0.99-1.10)	1.04	(0.99-1.10)
High middle (53.98 - 59.74)	0.99	(0.94-1.05)	0.99	(0.94-1.05)	0.97	(0.92-1.03)	0.97	(0.92-1.03)
Highest (≥59.75)	0.98	(0.93-1.04)	0.98	(0.93-1.04)	0.95	(0.89-1.00)	0.95	(0.89-1.00)
Population density (reference rural-agricultural)								
Semi-urban	1.07	(1.02-1.12)^b^	1.07	(1.02-1.13)^b^	1.10	(1.04-1.15)^c^	1.10	(1.04-1.15)^c^
Urban	1.10	(1.04-1.16)^c^	1.10	(1.05-1.16)^c^	1.11	(1.05-1.17)^c^	1.11	(1.05-1.17)^c^
Metropolitan	1.12	(1.06-1.18)^c^	1.13	(1.07-1.19)^c^	1.17	(1.10-1.24)^c^	1.17	(1.10-1.25)^c^
Intercept	0.52	(0.44-0.60)^c^	0.54	(0.46-0.63)^c^	0.50	(0.45-0.56)^c^	0.51	(0.46-0.58)^c^
Random effects								
Local district-level variance (SE)	0.000	0.000	0.000	0.000	0.000	0.000	0.000	0.000
Municipality-level variance (SE)	0.000	0.000	0.000	0.000	0.000	0.000	0.000	0.000

Null model for males: Intercept, multilevel PR: 0.68 (0.66 - 0.69), *p* < 0.001, local district-level variance (SE): 0.000 (0.000), municipality-level variance (SE): 0.002 (0.001).

Null model for females: Intercept, multilevel PR: 0.67 (0.66 - 0.69), *p* < 0.001, local district-level variance (SE): 0.000 (0.000), municipality-level variance (SE): 0.002 (0.001).

PR, prevalence ratio; CI, confidence interval; SE, standard error.

^a^, *p* < 0.05; ^b^, *p* < 0.01; ^c^, *p* < 0.001.

All local district-level social capital variables were not associated with denture/bridge use.

## Discussion

The results of the present study showed that factors independently associated with denture/bridge use in both sexes were equivalent income, number of teeth, present illness, and population density, all of which are known to be associated with access to dental care [7]. In particular, individual financial status was strongly associated with denture/bridge use in the present study, which is in agreement with findings of a study conducted in one municipality in Japan [15]. Studies have suggested that low socioeconomic status is one of the barriers to dental attendance and that such barriers appear to have negative effects on oral health [23,24]. It is noteworthy that even in people with universal free access to dental services under the national healthcare insurance system in Japan, financial issues are a major factor affecting denture/bridge use.

Subjects presently having illness were more likely to use a denture/bridge in the present study. Systemic ill health and functional limitations have been reported as barriers to seeking dental health care [25]. The results of the present study do not corroborate these findings. However, this discrepancy can be explained as follows. First, all subjects in the present study were ADL independent; therefore, functional limitations were not barriers to seeking dental health care in the present study. Second, subjects presently having illness may be more likely to ask dentists as well as doctors to solve their health problems, because a study using dental and medical care insurance records of employees aged 20–39 years showed that individuals who consulted dentists tended to receive medical treatment more frequently [26].

Although both sexes shared the same factors associated with denture/bridge use, there were differences in factors associated with denture/bridge use between sexes. The results of the present study showed that females involved in one or more kinds of social groups were more likely to use a denture/bridge. These results agree with an interview study from the UK that showed that patients focused on the social significance of oral rehabilitation when defining the need for a removable partial denture [16].

High educational attainment was associated with denture/bridge use only in males. Few studies have reported gender difference in the association between educational attainment and oral health status and/or oral health behavior, probably because most of the studies analyzed the association including both sexes [8-10,23,24]. A study in a Japanese older population showed that males with the highest educational attainment showed healthier ageing and lower mortality compared to males with the lowest educational attainment; however, no such differences were seen among females [27]. These results suggest that educational attainment is associated with oral and systemic health in males, but not in females. Further studies that confirm the reproducibility of these findings are needed to explain the gender difference.

No social capital variables were associated with denture/bridge use. These results disagreed with those from a recent study that suggested that older people living in societies with rich social capital tend to have good oral health status, including having 20 or more teeth [18]. The results of the present study suggest that denture/bridge use was associated with personal factors, such as financial and social factors, but not social capital.

In contrast to social capital, high population density was associated with denture/bridge use in the present study. Because population density may be considered as surrogate information on socioeconomic status, it is possible that people living in richer areas tend to use denture/bridge.

The results of the present study show that target groups in which percentage of people using denture/bridge must be increased included people with low income and those living in the area with low population density. In addition, males with low educational attainment and females who do not have any social groups should be targeted. Taking this information into consideration, formulation of an intervention program for the target groups is recommended from the public health perspective.

The present study had a number of limitations. First, denture use was not distinguished from bridge use in the present study, which makes it difficult to interpret the results. A bridge is a fixed prosthesis and cannot be removed by patients; however, a denture can be removed by patients, and denture use is thus affected by patient compliance. To partially address this issue, we excluded subjects with 20 or more teeth and added number of teeth as a variable in the analyses.

Second, we did not obtain information regarding dental implants which is another type of dental prosthesis because dental implant is not covered by public health insurance in Japan. A recent national survey showed that 4.4%, 1.2% and 2.8% of the whole population aged 65–74, 75–84 and 85- had dental implants, respectively [5]. Additional studies adding information on dental implants are necessary to confirm the results of the present study in the future.

Third, the state of the denture such as stability and fit was unknown because this study was based on a self-administered questionnaire. Our previous study using a similar self-administered questionnaire showed that 13.7% of the participants with few teeth and dentures reported poorly fitted dentures [2]. Additional studies are needed to confirm the results of the present study using information on status of dentures.

Fourth, the measurements used were based on a self-administered questionnaire. Some forms of bias, such as social desirability bias [28], may have affected the results of the present study. Fifth, because this was a cross-sectional study, causal relationships are unclear.

## Conclusions

Denture/bridge use was significantly associated with high economic status, present illness, and living in an area with high population density in both sexes among community-dwelling older Japanese having 19 or fewer teeth. Different factors were associated with denture/bridge use in males and females: high educational attainment in males and involvement in one or more social groups in females. Local district-level social capital was not associated with denture/bridge use.

## Abbreviations

JAGES: Japan gerontological evaluation study; ADL: Activities of daily living; PR: Prevalence ratio; CI: Confidence interval.

## Competing interests

The authors declare that they have no competing interests.

## Authors’ contributions

TY conceived the idea for the study, participated in its design, performed the statistical analysis and drafted the manuscript as the principal author. KK is the principal investigator of the JAGES project, helped to develop the idea of the study, participated in acquiring the data and the study design, and edited the manuscript. JA, KS, JM, and MN participated in data acquisition and study design and critically revised the manuscript. SF helped with data analysis and critically revised the manuscript. YH helped to develop the idea of the study, participated in the study design, and edited the manuscript. All authors read and approved the final manuscript.

## Pre-publication history

The pre-publication history for this paper can be accessed here:

http://www.biomedcentral.com/1472-6831/14/63/prepub
